# Home ownership, full-time employment, and other markers of higher socioeconomic status are predictive of shorter time to initial evaluation, shorter time to surgery, and superior postoperative outcomes among lateral patellar instability patients undergoing medial patellofemoral ligament reconstruction

**DOI:** 10.1186/s43019-023-00193-3

**Published:** 2023-07-17

**Authors:** Dhruv S. Shankar, Amanda Avila, Brittany DeClouette, Kinjal D. Vasavada, Isabella B. Jazrawi, Michael J. Alaia, Guillem Gonzalez-Lomas, Eric J. Strauss, Kirk A. Campbell

**Affiliations:** Department of Orthopedic Surgery, New York University Langone Health, 333 East 38Th St, 4Th Floor, New York, NY 10016 USA

**Keywords:** Patellar instability, MPFL, Socioeconomic, Patient-reported outcomes, Time to surgery, Return, To sport

## Abstract

**Background:**

The purpose of this study was to identify socioeconomic predictors of time to initial evaluation, time to surgery, and postoperative outcomes among lateral patellar instability patients undergoing medial patellofemoral ligament reconstruction (MPFLR).

**Methods:**

We conducted a retrospective review of patients at our institution who underwent primary MPFLR with allograft from 2011 to 2019 and had minimum 12-month follow-up. Patients were administered an email survey in January 2022 to assess symptom history, socioeconomic status, and postoperative outcomes including VAS satisfaction and Kujala score. Predictors of time to initial evaluation, time to surgery, and postoperative outcomes were identified using multivariable linear and logistic regression with stepwise selection.

**Results:**

Seventy patients were included in the cohort (mean age 24.8 years, 72.9% female, mean follow-up time 45.7 months). Mean time to evaluation was 6.4 months (range 0–221) and mean time to surgery was 73.6 months (range 0–444). Having a general health check-up in the year prior to surgery was predictive of shorter time to initial evaluation (β = − 100.5 [− 174.5, − 26.5], *p* = 0.008). Home ownership was predictive of shorter time to surgery (β = − 56.5 [− 104.7, 8.3], *p* = 0.02). Full-time employment was predictive of higher VAS satisfaction (β = 14.1 [4.3, 23.9], *p* = 0.006) and higher Kujala score (β = 8.7 [0.9, 16.5], *p* = 0.03).

**Conclusion:**

Markers of higher socioeconomic status including having a general check-up in the year prior to surgery, home ownership, and full-time employment were predictive of shorter time to initial evaluation, shorter time to surgery, and superior postoperative outcomes.

*Level of evidence*: IV, retrospective case series.

## Introduction

Lateral patellar instability is a relatively common source of knee-related disability among young athletes and disproportionately affects females in the second and third decades of life [31]. Untreated recurrent lateral patellar instability has been associated with intra-articular cartilage damage that may produce severe knee-related limitations in both daily and athletic activities [5, 19]. The common denominator of this condition is deficiency of the medial patellofemoral ligament (MPFL), which serves as the primary restraint to lateral patellar translation at low flexion angles [2]. Operative management of patellar instability has become increasingly popular over the past two decades and MPFL reconstruction (MPFLR) has been associated with satisfactory outcomes and a significant reduction in instability episodes [24, 25].

As in other fields of medicine, social determinants of health have a major impact on health literacy about musculoskeletal disorders, access to orthopedic care, and outcomes following orthopedic surgery [11, 23]. In the setting of patellar instability and operative treatment with MPFLR, certain socioeconomic characteristics may be of benefit or an obstacle to early recognition of patellar instability and seeking appropriate treatment for the condition. Prior analyses by Li et al. and Allahabadi et al. have found that race and insurance status may be significant predictors of access to surgical care, timing of initial evaluation and surgery, and cost of treatment among patellar instability patients [1, 16]. This is especially important given that chronicity of instability symptoms has been associated with increased likelihood of sequelae such as chondral lesions, chronic pain, and early osteoarthrosis [5, 19]. Therefore, identifying barriers to timely access to orthopedic care for patellar instability may enable surgeons to intervene at an earlier stage in the disease process when postoperative outcomes may be more favorable. Furthermore, a comprehensive analysis is needed to identify socioeconomic predictors besides race and insurance status such as household income and neighborhood socioeconomic status; these factors have been associated with treatment delays and postoperative outcomes in other sports medicine patient populations such as anterior cruciate ligament reconstruction (ACLR) patients [18, 26], but have thus far not been studied in patellar instability patients undergoing MPFLR.

The purpose of this study was to identify socioeconomic predictors of time to initial evaluation, time to surgery, and postoperative outcomes among lateral patellar instability patients undergoing MPFLR. Based on prior findings from the ACLR literature, we hypothesized that markers of higher socioeconomic status would be predictive of shorter time to initial evaluation, shorter time to surgery, and superior postoperative outcomes.

## Methods

### Study design and setting

We conducted a retrospective case series at a single urban academic medical center.

### Ethical approval

Ethical approval for this study was obtained from our institutional review board and a waiver of consent was granted.

### Cohort selection

We enrolled patients who underwent MPFLR with allograft for treatment of lateral patellar instability from January 2011 to December 2019 with one of five sports medicine fellowship-trained orthopedic surgeons at our center. Eligible patients were identified in the electronic medical record system using CPT codes 27405, 27420, 27422, 27424, 27425, 27427, and 27429. Inclusion criteria were (1) diagnosis of lateral patellar instability, (2) MPFLR surgery with or without tibial tubercle osteotomy (TTO), (3) skeletal maturity at the time of surgery, and (4) minimum follow-up of 12 months. Patients who underwent prior MPFLR and subsequently underwent revision MPFLR were excluded.

### Surgical indications

Open MPFLR was indicated for patients with recurrent patellar instability as defined by > 1 dislocation or subluxation event. Concomitant procedures among the cohort included anteromedialization TTO, arthroscopic shaving chondroplasty, and arthroscopic partial meniscectomy. Open TTO was performed in patients with radiographic evidence of patellofemoral malalignment as defined by a tibial tubercle-to-trochlear groove (TT-TG) distance > 15 mm on axial knee magnetic resonance imaging (MRI). MPFLR and TTO were performed as open procedures. Arthroscopic chondroplasty was performed for chondral lesions < 1 cm^2^ in area. Arthroscopic partial meniscectomy was performed for meniscal tears that were not amenable to repair (i.e. displaced, unstable, within the white-white zone).

### Chart review and radiographic data collection

Age at time of surgery, sex, insurance status at time of surgery (commercial or Medicaid), date of surgery, procedure laterality, MPFL graft type, and concomitant procedures were abstracted from electronic medical records. Preoperative knee imaging was obtained for all patients as part of the standard of care and included MRI scans with axial and sagittal slices and standing knee X-rays with anteroposterior (AP) and lateral views. For each index knee, tibial tubercle-to-trochlear groove (TT-TG) distance was measured on axial MRI, Dejour classification of trochlear dysplasia was assessed on both axial MRI and lateral X-ray, and patellar height was assessed using the Insall-Salvati (IS) ratio measured on sagittal MRI. Patella alta was defined as an IS ratio > 1.2 and patella baja was defined as an IS ratio < 0.8 and these cutoff values have been validated for both adult and pediatric populations [6, 14].

### Survey data collection

Patellar instability symptom history, socioeconomic factors, and postoperative outcomes were assessed using an email survey distributed using the REDCap data capture system [7, 8]. Surveys were distributed on January 3^rd^, 2022 and survey responses were solicited through February 4th, 2022. Follow-up time was defined as the time elapsed between the date of surgery and date of survey response.

#### Symptom history, time to initial evaluation, and time to surgery

Patients reported whether their patellar instability symptoms were of traumatic or atraumatic onset, whether they experienced patellar subluxation at least once a week prior to surgery, and whether they experienced at least one dislocation episode prior to surgery. Patients with traumatic-onset instability reported their date of injury and patients with atraumatic-onset instability reported their date of symptom onset. Patients also reported their date of initial evaluation by a physician for patellar instability symptoms; this physician did not need to be from the study institution. When possible, electronic medical records were used to corroborate patient-reported dates of injury, symptom onset, and initial evaluation. Time to initial evaluation was calculated as the time elapsed between the date of injury/symptom onset and the date of initial evaluation. Time to surgery was calculated as the time elapsed between the date of injury/symptom onset and the date of surgery.

#### Socioeconomic status

Socioeconomic variables were adapted from 2012 guidelines proposed by the U.S. National Committee on Vital and Health Statistics (NCVHS) [21] and included the following: first spoken language, self-identified race, Hispanic or Latino ethnicity, marital status, household size, annual household income, home ownership, vehicle ownership, whether or not the patient took an out-of-town vacation in the year prior to surgery, highest level of education completed, intent to pursue further education, employment status, occupation type, and whether the patient had seen a physician for a general check-up in the year prior to surgery. Patients were asked to report all socioeconomic characteristics based on their status prior to surgery.

The socioeconomic status of each patient’s neighborhood was quantified using the University of Wisconsin School of Medicine and Public Health U.S. Area Deprivation Index (ADI) [12, 27] and the Centers for Disease Control/Agency for Toxic Substances and Disease Registry (CDC/ATSDR) Social Vulnerability Index (SVI) [4]. The former metric ranks neighborhoods’ socioeconomic status on the basis of income, education, employment, and housing quality while the latter ranks neighborhoods’ socioeconomic status on the basis of vulnerability to natural or human-made disaster events (ex. hurricanes, chemical spills). Patients’ home ZIP codes at the time of surgery were obtained from electronic medical records and codes were matched to their corresponding ADI and SVI. For both metrics, national percentiles were used in order to compare neighborhood socioeconomic status among patients from different states within our cohort.

#### Postoperative outcomes

Postoperative outcomes included reoperations, recurrent patellar instability, 10-point Visual Analog Scale (VAS) knee pain at rest, 10-point VAS knee pain during sports/physical activity, 100-point VAS satisfaction with the index procedure [28–30], Kujala Anterior Knee score, MPFL-Return to Sport after Injury (MPFL-RSI) score, return to sport, and return to work. For the MPFL-RSI instrument, a score > 56 was considered a passing score indicating psychological readiness to return to sport [10]. For return to sport outcomes, preoperative sport participation was classified as either contact or non-contact. For return to work outcomes, preoperative work intensity was classified using the Association for Work Design, Business Organization and Business Development (REFA, for its acronym in German) workload classification system [13]. Workload classifications ranged from Grade 0 (“work without special physical strain”) to Grade 4 (“work with most heavily physical strain”). Patients were asked to report all postoperative outcomes based on their status at the time of survey completion.

### Statistical analysis

All statistical analyses were performed in SAS Studio version 9.4 (SAS Institute, Cary, NC, USA). Descriptive statistics were calculated for all variables (mean and standard deviation for continuous variables, count and percentage for categorical variables). All continuous variables were assessed for normality using the Shapiro–Wilk test. Inter-group comparisons of non-normally distributed continuous variables were performed using the Mann–Whitney *U* test. Inter-group comparisons of categorical variables were performed using Fisher’s exact test. Demographic, clinical, and socioeconomic predictors of time to initial evaluation, time to surgery, and continuous postoperative outcomes (ex. VAS pain, Kujala score) were identified using multiple linear regression. Predictors of binary outcomes (ex. recurrent instability, passing MPFL-RSI score) were identified using multivariable logistic regression. For both types of models, all demographic, socioeconomic, and perioperative variables were entered as potential predictors. Interaction terms between socioeconomic variables (ex. race and income, income and college education) were also included as potential predictors. Selection of predictors was performed using stepwise selection with the aim of minimizing the Bayesian information criterion for each outcome model. After significant predictors were selected, linear/logistic regression was performed again using the selected variables while adjusting for age as a co-predictor. For each predictor variable, beta (β) coefficients from linear regression models and odds ratios (Ors) from logistic regression models were calculated with 95% confidence intervals and *p*-values. Subgroup analyses were performed to identify predictors of outcomes among patients undergoing MPFLR without concomitant TTO and those undergoing MPFLR with TTO. P-values < 0.05 were considered significant.

## Results

### Cohort demographics

Seventy patients were included in the final cohort (Table 1). The mean age of the cohort was 24.8 years (range 12–56), most patients were female (51 patients; 72.9%), and mean follow-up time was 45.7 months (range 12–108). About half of patients had patellar instability of traumatic etiology (36 patients; 51.4%). The majority of the cohort identified as white (48 patients; 68.6%). About half had completed a 4-year college degree (33 patients; 47.2%) and had an annual household income of $100,000 or more (28 patients; 45.2%). By occupation type, the largest groups were students (27 patients; 38.6%) followed by patients in service occupations (14 patients; 20.0%). Most patients were on commercial insurance at the time of surgery (65 patients; 92.9%) and had a general health check-up within the year prior to their surgery (61 patients; 87.1%).

**Table 1:** Patient demographics and socioeconomic characteristics

Variable	Count (%) or mean ± standard deviation
N	70
Demographics and symptom history	
Age (years)	24.8 ± 9.2
Follow-up time (months)	45.7 ± 25.3
Sex	Male 19 (27.1%)|Female 51 (72.9%)
Traumatic injury	36 (51.4%)
Subluxations occurring at least once a week	22 (31.4%)
At least one dislocation episode prior to surgery	24 (34.3%)
Socioeconomic characteristics	
Insurance type	Commercial 65 (92.9%)|Medicaid 5 (7.1%)
English first language	67 (95.7%)
Race	White 48 (68.6%) Black or African–American 7 (10.0%) Asian 6 (8.6%) Native American, American Indian, or Alaska Native 1 (1.4%) Native Hawaiian or Other Pacific Islander 1 (1.4%) Other Non-White Race 7 (10.0%)
Hispanic or Latino ethnicity	9 (12.9%)
Marital status	Never married 56 (80%)|Married 13 (18.6%) | Separated 1 (1.4%)
Household size ≥ 3 people	28 (40.0%)
Annual household income	Less than $5000 8 (12.9%) $5000–$29,999 5 (8.1%) $30,000–$74,999 13 (8.1%) $75,000–$99,999 8 (12.9%) $100,000–$124,999 10 (16.1%) $125,000–$149,999 6 (9.7%) $150,000 or more 12 (19.4%)
Home ownership (including payments on a mortgage)	41 (58.6%)
Vehicle ownership	50 (71.4%)
Took an out-of-town vacation in the year prior to surgery	57 (81.4%)
Highest level of education	Didn't finish high school 12 (17.1%) Some high school and technical/vocational program 0 (0.0%) High school graduate or GED 13 (18.6%) Completed high school and technical/vocational program 2 (2.9%) Less than 2 years of college 6 (8.6%) 2 years of college or more 4 (5.7%) College graduate 21 (30.0%) Master's degree 10 (14.3%) Doctoral degree 2 (2.9%)
Pursuing further education	43 (61.4%)
Employed full time	29 (41.43%)
Occupation type	Unemployed 8 (11.4%) Student 27 (38.6%) Blue-collar occupation 3 (4.3%) Service occupation 14 (20.0%) Professional occupation 11 (15.7%) Managerial occupation 7 (10.0%)
Had general health check-up in year prior to surgery	61 (87.1%)

### Radiographic characteristics

Patellofemoral radiographic characteristics among the cohort are presented in Table 2. Most patients had a TT-TG distance > 15 mm on axial MRI (45 patients; 64.3%). A slight majority of the cohort had patella alta (40 patients; 57.1%) and no patients had patella baja. Most patients had trochlear dysplasia of Dejour types A and B (43 patients; 61.4%).

**Table 2:** Radiographic and operative information

Variable	Count (%) or mean ± standard deviation
N	70
Radiographic characteristics	
TT-TG distance on MRI (mm)	17.2 ± 3.9
TT-TG distance > 15 mm	45 (64.3%)
Insall-Salvati ratio on MRI	1.2 ± 0.2
Patellar height	Normal height 30 (42.9%) Patella alta 40 (57.1%)
Dejour classification of trochlear dysplasia	Type A 24 (34.3%) Type B 19 (27.1%) Type C 13 (18.6%) Type D 14 (20.0%)
Treatment course and operative information	
Time from symptom onset to initial evaluation (months)	6.4 ± 27.5
Time from symptom onset to surgery (months)	73.6 ± 104.7
Procedure laterality	Left 32 (45.7%)|Right 38 (54.3%)
Graft type	Gracilis 65 (92.9%)|Semitendinosus 5 (7.1%)
Had concomitant procedure	45 (64.3%)
Tibial tubercle osteotomy	36 (51.4%)
Meniscectomy	2 (2.9%)
Chondroplasty	30 (42.9%)
Loose body removal	10 (14.3%)

*TT-TG* tibial tubercle-to-trochlear groove, *MRI* magnetic resonance imaging

### Treatment course and operative characteristics

There was considerable variation among the cohort in time to initial evaluation and time to surgery (Table 2). Mean time to evaluation was 6.4 months (range 0–221) and mean time to surgery was 73.6 months (range 0–444). Almost all patients underwent MPFLR with gracilis allograft (65 patients; 92.9%) and a majority also had at least one concomitant procedure performed (45 patients; 64.3%), most commonly TTO (36 patients; 51.4%) and chondroplasty (30 patients; 42.9%).

### Complications and outcomes

Reoperation rates were low among the cohort (Table 3). Seven patients (10.0%) underwent reoperations, most commonly manipulation under anesthesia (4 patients; 5.7%). Of those who participated in sports prior to surgery (28 patients; 40.0%), 13 (46.4%) returned to sports after surgery. Of those who were employed prior to surgery (38 patients; 56.7%), 35 (92.1%) returned to work after surgery. Ten patients (14.7%) reported recurrent patellar instability following surgery. About half of the cohort (39 patients; 55.7%) achieved a passing score on the MPFL-RSI.

**Table 3:** Reoperations and postoperative outcomes

Variable	Count (%) or mean ± standard deviation
N	70
Reoperations	7 (10.0%)
Revisions	1 (1.4%)
Removal of hardware	1 (1.4%)
Manipulation under anesthesia	4 (5.7%)
Total knee arthroplasty	1 (1.4%)
Participated in sports before surgery	28 (40.0%)
Preoperative sport level of contact	Non-contact 14 (50.0%) Limited contact 13 (46.4%) Full contact 1 (3.6%)
Returned to sports	13 out of 28 (46.4%)
Returned to sports at the pre-injury level of competition	8 out of 28 (28.6%)
Employed before surgery	38 (56.7%)
Preoperative REFA class of work intensity	Class 0 35 (92.1%) Class 1 2 (5.3%) Class 2 1 (2.6%)
Returned to work	35 out of 38 (92.1%)
Postoperative recurrent instability	10 (14.7%)
VAS pain at rest (0–10 max)	1.2 ± 1.8
VAS pain during sports (0–10 max)	2.3 ± 2.5
VAS satisfaction (0–100 max)	84.2 ± 14.7
Kujala score (0–100 max)	83.9 ± 14.7
MPFL-RSI score (0–100 max)	60.1 ± 24.6
MPFL-RSI score > 56	39 (55.7%)

*REFA* Association for Work Design, Business Organization and Business Development, *VAS* visual Analog Scale, *MPFL-RSI* Medial Patellofemoral Ligament-Return to Sport after Injury

### Socioeconomic predictors of treatment course

We identified significant socioeconomic predictors for time to initial evaluation and time to surgery, independent of age (Table 4). Having a general check-up in the year prior to surgery was predictive of shorter time to initial evaluation (β = − 100.5, *p* = 0.008). Home ownership was predictive of shorter time to surgery (β = − 56.5, *p* = 0.02). Besides socioeconomic factors, preoperative patellar subluxation frequency of once a week or greater was predictive of both longer time to initial evaluation and longer time to surgery (*p* < 0.05).

**Table 4:** Significant predictors of time to initial evaluation, time to surgery, and postoperative outcomes

Outcome	Significant predictors^a^	β or OR with 95% CI	*p*-value^b^
Time to initial evaluation	Subluxations occurring at least once a week	β = 57.1 [3.9 to 110.2]	0.04
	Had general health check-up in year prior to surgery	β = − 100.5 [− 174.5 to − 26.5]	0.008
Time to surgery	Subluxations occurring at least once a week prior to surgery	β = 58.3 [7.1 to 109.5]	0.03
	Home ownership	β = − 56.5 [− 104.7 to − 8.3]	0.02
Reoperation	Age	OR = 1.1 [1.0 to 1.2]	0.02
	At least one dislocation episode prior to surgery	OR = 10.3 [1.4 to 76.7]	0.02
Postoperative recurrent instability	Subluxations occurring at least once a week prior to surgery	OR = 8.7 [1.4 to 53.5]	0.02
	Home ownership	OR = 0.1 [0.0 to 0.6]	0.01
	Employed full-time	OR = 0.1 [0.0 to 0.8]	0.03
Return to sport	No significant predictors	n/a	n/a
Return to work	No significant predictors	n/a	n/a
VAS pain	Concomitant procedure(s)	β = 9.0 [0.2 to 17.8]	0.045
VAS sports	No significant predictors	n/a	n/a
VAS satisfaction	Employed full-time	β = 14.1 [4.3 to 23.9]	0.006
	Non-white race AND at least 2 years of college education	β = − 18.3 [− 35.1 to − 1.6]	0.03
	Non-white race AND home ownership	β = 42.6 [23.6 to 52.6]	< 0.001
	Non-white race AND took at least one vacation	β = − 23.4 [− 42.2 to − 4.6]	0.02
Kujala score	Age	β = − 0.6 [− 1.0 to − 0.4]	0.004
	Employed full-time	β = 8.7 [0.9 to 16.5]	0.03
MPFL-RSI score	No significant predictors	n/a	n/a
MPFL-RSI > 56	No significant predictors	n/a	n/a

^a^All predictors are adjusted for age at the time of surgery

^b^All *p*-values are < 0.05

*TT-TG* tibial tubercle-to-trochlear groove, *TTO *tibial tubercle osteotomy, *VAS* Visual Analog Scale, *MPFL-RSI* Medial Patellofemoral Ligament-Return to Sport After Injury

### Socioeconomic predictors of postoperative outcomes

We identified significant socioeconomic predictors of postoperative outcomes (Table 4). Home ownership and full-time employment were predictive of lower odds of having postoperative recurrent patellar instability (*p* < 0.05). Full-time employment was predictive of greater VAS satisfaction (β = 14.1, *p* = 0.006). Non-white race was not an independent predictor of VAS satisfaction (*p* > 0.05). However, the interactions of non-white race with (1) having at least two years of college education and (2) taking with at least one vacation in the year prior to surgery were predictive of lower VAS satisfaction (*p* < 0.05). Conversely, the interaction of non-white race with home ownership was predictive of greater VAS satisfaction (β = 42.6, *p* < 0.001). Full-time employment was predictive of higher Kujala score (β = 8.7, *p* = 0.03). There were no significant socioeconomic predictors of the odds of undergoing a reoperation, return to sport, return to work, VAS pain at rest, VAS pain with sports, MPFL-RSI score, or the odds of scoring > 56 on the MPFL-RSI (all *p* > 0.05).

### MPFLR with versus without TTO

Thirty-four patients underwent MPFLR without TTO and 36 patients underwent MPFLR with TTO. At baseline, there were no significant differences in age (*p* = 0.10), sex (*p* = 0.79), preoperative incidence of patellar dislocation (*p* = 0.21), follow-up time (*p* = 0.21), or socioeconomic characteristics (*p* > 0.05). However, a higher percentage of patients in the non-TTO group had traumatic-onset instability (64.7% vs. 38.9%, *p* = 0.04) whereas a higher percentage of patients in the TTO group had preoperative subluxation frequency of once a week or greater (14.7% vs. 47.2%, *p* = 0.005). A higher percentage of the TTO group had TT-TG distance > 15 mm (35.3% vs. 91.7%, *p* < 0.001).

The non-TTO group had significantly shorter time to initial evaluation (2.7 vs. 47.5 months, *p* = 0.048) and significantly shorter time to surgery (46.1 vs. 99.5 months, *p* = 0.002). The TTO group had a higher incidence of postoperative recurrent patellar instability (3.0% vs. 25.7%, *p* = 0.01). There were no significant inter-group differences in incidence of reoperations (*p* = 0.71), return to sport (*p* = 0.71), return to work (*p* = 0.23), postoperative VAS pain at rest (*p* = 0.75), VAS pain with sports (*p* = 0.39), VAS satisfaction (*p* = 0.96), Kujala score (*p* = 0.50), or MPFL-RSI score (*p* = 0.61).

In the non-TTO group, annual household income > $75,000 was predictive of shorter time to initial evaluation (β = − 37.4 [− 50.5, − 24.3], *p* < 0.001) and having at least two years of college education was predictive of lower postoperative VAS pain at rest (β = − 16.2 [− 30.3, − 2.1], *p* = 0.03).

In the TTO group, predictors of shorter time to initial evaluation were annual household income > $75,000 (β = − 94.6 [− 181.2, − 7.9], *p* = 0.03) and having a general health check-up in the year prior to surgery (β = − 279.1 [− 398.9, − 159.4], *p* < 0.001). Predictors of shorter time to surgery included vehicle ownership (β = − 156.7 [− 296.4, − 17.0], *p* = 0.03) and being of white race with annual household income > $75,000 (β = − 179.8 [− 322.4, − 37.1], *p* = 0.02). With regard to postoperative outcomes, full-time employment was predictive of higher VAS satisfaction (β = 10.2 [0.3, 20.2], *p* = 0.04) and being married was predictive of higher Kujala score (β = 25.3 [8.4, 42.1], *p* = 0.005).

## Discussion

Having a general health check-up in the year prior to surgery was predictive of shorter time to initial evaluation for patellar instability and home ownership was predictive of shorter time to MPFLR. Furthermore, home ownership and full-time employment, both markers of higher socioeconomic status, were predictive of some superior outcomes including lower odds of postoperative recurrent instability, higher VAS satisfaction, and higher Kujala score. Race was found to be both a positive and negative predictor of postoperative satisfaction in interaction with other socioeconomic markers including level of college education, home ownership, and out-of-town vacation time. Overall, our findings support our hypothesis that markers of higher socioeconomic status would be predictive of shorter time to initial evaluation, shorter time to surgery, and superior postoperative outcomes.

Unlike other knee ligamentous pathologies such as ACL injury, patellar instability secondary to MPFL deficiency is often a chronic condition that presents in adolescence and is marked by recurrent symptoms that may worsen over time [1]. Given the adverse sequelae (e.g. chondral injury, osteoarthrosis) associated with chronic patellar instability [5, 19], timely evaluation and surgical treatment for instability symptoms can improve knee-related quality of life. Conversely, delays in evaluation and treatment could portend more severe pathology and worse postoperative outcomes. The chronicity of patellar instability and the potential impact of time to evaluation/surgery was the impetus for prior studies by Li et al. [16] and Allahabadi et al. [1], who sought to identify socioeconomic barriers to surgical access in this unique segment of the broader sports medicine patient population.

The impact of race on patellar instability treatment and MPFLR outcomes has been sparsely discussed in the orthopedic literature. While the majority of pediatric hospitalizations for patellar instability between 2004 and 2017 occurred among white patients [20], some studies have noted racial disparities that negatively impact minority groups. Waterman et al. previously identified racial disparities in the incidence of patellar dislocation in a large cohort drawn from the National Electronic Injury Surveillance System (NIESS) database and found a significantly higher incidence among white and black patients compared to Hispanic patients [31]. However, the authors also noted a significant limitation of this finding that only 67% of their dataset had a documented racial category. Similarly, Hsiao et al. identified a higher incidence of patellar dislocation among white and black patients compared to patients categorized as “Other Race” in a cohort of active-duty U.S. service members, but their analysis was also limited by the heterogeneity of the “Other Race” category [9]. Racial disparities have also been identified in the operative treatment of patellar instability. Li et al. found that black pediatric patients had lower odds of undergoing surgery and higher charges for care compared to their white counterparts, based on an analysis of a large cohort drawn from the New York Statewide Planning and Research Cooperative System (SPARCS) database [16]. In a single-center retrospective case series, Allahabadi et al. [1] found that public insurance status was a significant predictor of longer time to initial evaluation, MRI, and surgery. Our own analysis demonstrated a complex relationship between race and satisfaction following MPFLR, with the effect of race being modified by other socioeconomic variables such as education status and home ownership. As such, the interaction of race with social determinants of health cannot be overlooked and future analyses of racial differences in the presentation of patellar instability and postoperative outcomes of MPFLR must account for these interactions.

Our analysis also identified home ownership and full-time employment to be predictive of a number of positive outcomes related to patellar instability treatment, including shorter time to surgery, lower odds of postoperative instability, and higher satisfaction and Kujala scores. While home ownership has been long been recognized in the social sciences literature as a key determinant of socioeconomic status, with the home serving as both an asset in itself and as a means to acquire further wealth [32], it has not been as extensively discussed in the orthopedics literature on social determinants of health. Of note, Callahan et al. found home ownership, alongside occupation, educational attainment, income, and community poverty, to be an independent predictor of physical functioning, functional disability, and health-related quality of life among a cohort of 968 adults with arthritis [3]. However, no similar analysis has been performed in the patellar instability population. By comparison, employment has been more widely discussed as a predictor for outcomes in the orthopedics literature, though typically in the context of the level of physical activity required for a given occupation rather than as a marker of socioeconomic status. Full-time employment may confer a number of benefits for patients receiving orthopedic care, such as access to employer-sponsored health insurance plans that cover subspecialty services as well as paid sick leave which may be used during the postoperative rehabilitation period [17]. Consequently, the association between employment status and postoperative outcomes observed in our study may reflect patients’ access to financial resources to cover the cost of surgery and rehabilitation during a period when they may be physically unable to perform work.

Our subgroup analysis comparing patellar instability patients who underwent MPFLR with versus without TTO identified several socioeconomic predictors of treatment course and MPFLR outcomes among both groups. In both groups, annual household income > $75,000 was associated with shorter time to initial evaluation. However, the analysis of the TTO group yielded significant more socioeconomic predictors of time to surgery and postoperative outcomes than the analysis of the non-TTO group. This discrepancy may stem from the fact that a significantly higher percentage of patients in the non-TTO group had patellar instability of traumatic onset. In the setting of trauma, patients may have sought more urgent treatment for their symptoms regardless of socioeconomic barriers. This is evidenced by the significantly shorter time to evaluation and surgery among the non-TTO group compared to the TTO group. Nonetheless, in both groups, markers of higher socioeconomic status (ex. greater household income, vehicle ownership, being married) were associated with shorter time to initial evaluation, shorter time to surgery, and/or superior postoperative outcomes.

Patellar instability patients could clinically benefit from social interventions aimed at addressing socioeconomic risk factors for delayed evaluation/treatment and poor postoperative outcomes. As suggested by our study, general health check-up habits are a strong predictor of seeking timely evaluation for patellar instability symptoms. Compliance with annual check-ups is often considered a function of health literacy [15]; system-level interventions that educate vulnerable patient populations about the importance of early screening for musculoskeletal disorders could potentially help to reduce the time to initial evaluation for patellar instability. At the individual level, sports medicine surgeons can aim to form close working relationships with primary care providers to simplify referrals and streamline access to specialty care for patellar instability symptoms. Our study also suggests that patients who do not own their home and are not employed full-time may be more likely to have symptom recurrence, have worse satisfaction, and have more severe patellofemoral dysfunction. These markers of lower socioeconomic status may be associated with a lack of access to other resources (ex. paid time off, insurance coverage) needed to facilitate recovery and rehabilitation following MPFLR surgery. Aside from system-level interventions to reduce financial barriers to orthopedic care (ex. Medicaid reform) [22], surgeons can help address these barriers by directly discussing financial barriers to postoperative care and rehabilitation with their patients and providing referrals to community resources to assist with medical bill coverage and paid/sick leave.

We note several limitations of our study design. First, the retrospective nature of the study may have introduced selection bias into our cohort. Second, our cohort does not include significant representation of patients on public insurance, which may be a direct consequence of our institution’s status as a private academic medical center. Third, we grouped all non-white races into a single category for the purposes of our analysis, and thus we were unable to identify differences in outcomes between specific minority groups compared to their white counterparts. Fourth, while some patients began experiencing patellar instability after an acute dislocation or another inciting incident, other patients experienced gradual onset of symptoms. Patients in the latter category may not have been able to accurately recall the date of their symptom onset and this could have introduced bias into the analysis of time to initial evaluation and time to surgery. Fifth, our inclusion criteria stipulated that patients needed to have minimum 12-month follow-up. However, this criterion may have skewed our results by excluding some patients of lower socioeconomic status who may not have had the resources to access postoperative care (ex. physical therapy) or follow up appropriately. Sixth, the use of stepwise selection in our regression models could have resulted in inflated values for regression coefficients and odds ratios. We caution that our regression results are indicative of the direction but not the magnitude of the relationship between each predictor variable and its corresponding outcome variable. Seventh, the sample size of this study was small and potentially unrepresentative of the broader patellar instability patient population, which raises the possibility of the hasty generalization fallacy affecting the validity of our findings. Given this, we caution that our analysis only identified statistical (not causative) relationships between specific socioeconomic factors and treatment outcomes, and that these results should be considered preliminary until supported with a larger cohort. Nonetheless, our findings may serve as a starting point for future analyses that address the same topic.

## Conclusion

Markers of higher socioeconomic status including having a general check-up in the year prior to surgery, home ownership, and full-time employment were predictive of shorter time to initial evaluation, shorter time to surgery, and superior postoperative outcomes.

## Data Availability

The datasets used and/or analyzed during the current study are available from the corresponding author on reasonable request.

## Abbreviations

ADI: Area Deprivation Index
AP: Anteroposterior
CDC/ATSDR: Centers for Disease Control/Agency for Toxic Substances and Disease Registry
IS: Insall-Salvati
MPFL: Medial patellofemoral ligament
MPFLR: Medial patellofemoral ligament reconstruction
MPFL-RSI: Medial patellofemoral ligament-return to sport after injury
MRI: Magnetic resonance imaging
NCVHS: National Committee on Vital and Health Statistics
NIESS: National Electronic Injury Surveillance System
OR: Odds ratio
REFA: Association for Work Design, Business Organization and Business Development
ROC: Receiver operating characteristic
SPARCS: Statewide Planning and Research Cooperative System
SVI: Social Vulnerability Index
TTO: Tibial tubercle osteotomy
TT-TG: Tibial tubercle-to-trochlear groove
VAS: Visual Analog Scale
